# Platelet concentrates in macular hole surgery. A journey through the labyrinth of terminology, preparation, and application: a comprehensive review

**DOI:** 10.1007/s00417-023-06365-x

**Published:** 2024-01-25

**Authors:** Francesco Gelormini, Sergio D’antico, Federico Ricardi, Guglielmo Parisi, Enrico Borrelli, Paola Marolo, Fabio Conte, Marika Salafia, Michele Reibaldi

**Affiliations:** 1 Department of Surgical Sciences, Eye Clinic Section, University of Turin, 10122 Turin, Italy; 2 Blood Bank, A.O.U. Città della Salute e della Scienza, Turin, Italy

**Keywords:** Review, Platelet concentrates, Macular hole surgery, Platelet concentrates terminology

## Abstract

The surgical management of macular holes is undergoing continuous evolution, with recent focus on the utilization of platelet concentrates as a promising adjunctive intervention. Currently, they present a valid surgical approach for achieving anatomical and functional success with a non-inferiority comparably to the alternative surgical techniques. Nonetheless, the utilization of varied platelet concentrates terminologies, coupled with the lack of standardization in their preparation methodologies, engenders both lexical confusion and challenges in comparing scientific studies published up until now. In this review, we summarized the published evidence concerning the application of platelet concentrates in macular holes surgery, aiming to clarify the terminology and methodologies employed and to establish a common consensus facilitating further development and diffusion of this promising technique.


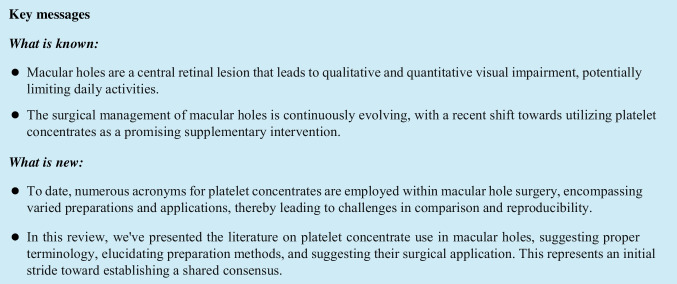


## Introduction

Full-thickness macular holes (FTMH) are retinal defects extending through the entire thickness of the retina, located at the center of the fovea, the central region of the retina responsible for sharp and detailed vision [1]. It is estimated that their prevalence in the general population is 3.3 per 1000 individuals [2], with a higher incidence in females and individuals aged 65 years and older [3].

Based on their etiology, they can be divided into primary or idiopathic FTMH (iFTMH), persistent/refractory FTMH (not closing after initial surgery), recurrent FTMH (closed and subsequently reopened after prior surgery), and secondary FTMH (caused by various underlying conditions such as myopia, trauma, retinal detachment, and macular telangiectasia) [2, 4].

Conversely, when the retinal defect identified through OCT includes an irregular foveal contour and a partial thickness defect (between inner and outer retinal layers) with preserved or interrupted outer retinal layers, it is referred to as a lamellar macular hole (LMH) [4].

The therapeutic strategies for addressing FTMH and LMH have continuously evolved over time.

In the case of LMH, both the surgical approach and the optimal timing for surgery remain subjects of ongoing debate. Some authors propose performing pars plana vitrectomy (PPV) with epiretinal membrane (ERM) and internal limiting membrane (ILM) peeling, followed by gas tamponade [5, 6], while others recommend solely performing ERM/ILM peeling [7, 8] or executing the double inverted ERM/ILM flap technique [9].

Regarding FTMHs, currently, a 25/27 Gauge PPV, combined with ILM peeling and gas tamponade, stands as the gold standard technique, ensuring a high macular hole (MH) closure rate (85–100%)[10], and postoperative best-corrected visual acuity (BCVA) improvement (58.3% at 52 weeks) [11, 12].

Diverse techniques for ILM peeling have been proposed [13], such as foveal sparing [14], complete ILM peeling [15], and ILM flap techniques (inverted flap [16], temporal inverted flap [17], free flap [18]). The latter demonstrated an increased MH closure rate in holes > 400 microns [16] and in recurrent/refractory/secondary FTMHs compared to ILM peeling alone [19], with a closure rate ranging from 80 to 100% [12, 16, 18, 20, 21].

Recently, particularly in the context of large/recurrent/refractory/secondary FTMHs, various forms of retinal plugs have been proposed as potentially promising interventions [22]. These include the lens capsular flap (MH closure rate 75–100%) [19, 23, 24], human amniotic membrane patch (hAM) (MH closure rate 57.1–100%) [25, 26], autologous retinal transplantation (MH closure rate 66.7–100%) [19, 27], macular hydrodissection (MH closure rate 83.3–100%) [19], and autologous plasma adjuvant treatment.

The therapeutic approaches for managing FTMH and LMH have undergone continuous evolution, with a recent focus on autologous plasma adjuvant treatment as a potentially promising intervention. The development and utilization of platelet concentrate as a surgical adjuvant to promote local healing represents a significant area of research applicable across diverse medical disciplines, with particular relevance in ophthalmology.

Platelets act as a natural reservoir of growth factors, including vascular endothelial growth factor (VEGF), platelet-derived growth factor (PDGF), and epidermal growth factor (EGF) [28]. Upon encountering altered or injured tissue, such as the retinal layers of a MH, these growth factors are released by platelets. Consequently, they could play a crucial role in the regeneration of macular defects [29]. Moreover, the presence of a fibrin clot following platelet activation could enhance tissue healing and act as a scaffold to facilitate the migration and cellular proliferation of Müller cells [29–31].

Therefore, the prospective therapeutic advantages of platelet concentrates (PCs) have prompted their increasing incorporation as an adjunctive approach in MH surgery. By modulating the processes of wound healing and tissue remodeling, this approach aimed to improve anatomical and visual outcomes for patients.

Following the widespread adoption of this surgical technique and the numerous evidence of its effectiveness, several research groups have published their results, using different terminology and different methodologies for preparing PCs. The common characteristic of all these adjuvants was that they were platelet concentrates, meaning they have a concentration of platelets that is by definition higher than that of whole blood. As clearly defined by Ehrenfest et al. [32], PRP is a generic term used to indicate PC, without considering differences in consistency and composition. Some researchers, including Choukroun et al. [33], used Platelet-Rich Fibrin (PRF) and considered it a second-generation PRP, despite PRF being a solid material, a blood clot, and not an injectable liquid solution like PRP. Furthermore, Choukroun’s PRF also contains leukocytes.

More recently, Mourao introduced a novel injectable form of PRF through a brief centrifugation process that initiates coagulation without completion, enabling the temporary utilization of liquid (injectable) PRF [34].

Another confounding factor in the development of a common terminology has been the use of the term “Platelet-Rich Plasma gel” (PRP gel), which refers to an activated form of PRP following contact with an activator substance such as calcium or thrombin. PRP gel is composed of an active matrix of platelet-rich fibrin and other growth factors, with a composition different from PRF [35]. Indeed, such terminology may inadvertently suggest that the 'standard' version of PRP is inactive and less effective, while in reality, the activation of the coagulation cascade occurs subsequently upon contact with the damaged tissue. Indeed, liquid PRP is generated by promptly adding an anticoagulant to the blood sample to halt the coagulation cascade, enabling the activation of coagulation and the formation of the platelet plug only after its injection. Conversely, to create PRF, immediate centrifugation is performed without the addition of an anticoagulant, allowing the natural formation of the clot. Based on these considerations, four categories of PCs can be defined: two types of PRP (P-PRP and L-PRP) and two types of PRF (P-PRF and L-PRF): (1) “Pure Platelet-Rich Plasma” (P-PRP) without leukocytes, (2) “Leukocyte and Platelet-Rich Plasma” (L-PRP) with leukocytes, (3) “Pure Platelet-Rich Fibrin” (P-PRF) without leukocytes, and (4) “Leukocyte and Platelet-Rich Fibrin” (L-PRF) with leukocytes [32]. The activated form of P-PRP and L-PRP is referred to as P-PRP gel and L-PRP gel, respectively, to highlight the difference in consistency compared to standard PRP and the different morphology of the fibrin matrix compared to PRF.

The purpose of this review is to summarize the evidence published regarding the use of PCs in MH, providing clarity on the terminology and methodology employed to establish a common consensus and facilitate further development and diffusion of this promising technique.

## Methodology

### Search methods

We conducted an extensive literature search using the PubMed, Medline, and Embase databases, covering the period from January 1993 to July 2023. The search terms utilized encompassed various relevant terms such as “platelet-rich plasma,” “autologous platelet concentrate,” “autologous thrombocyte serum concentrate,” “pure platelet-rich plasma,” “pure platelet-rich fibrin,” “liquid PRP,” “solid PRP,” “autologous platelets,” “autologous plasma,” “autologous adjuvants,” “plasma rich in growth factors,” “platelets concentrate,” and “autologous platelet concentrate” combined with “macular hole,” “idiopathic macular hole,” “recurrent macular hole,” “persistent macular hole,” “secondary macular hole,” “high myopic macular hole,” “refractory macular hole,” “traumatic macular hole,” “chronic macular hole,” “lamellar macular hole.”

### Article selections

All articles combined with the previously described keywords underwent scrutiny by two reviewers (F.G., F.R.). In instances of uncertainty, resolution entailed deliberations between the two reviewers and a third reviewer (G.P.). Articles written in a language other than English were excluded and duplicate articles were removed. Abstracts of unpublished studies were not included.

The inclusion criteria for the review were: retrospective and prospective articles investigating the use of PC in LMH and FTMH of any size or etiology (iFTMH, persistent/refractory FTMH, recurrent FTMH, and secondary FTMH). Exclusion criteria encompassed studies lacking detailed descriptions of the surgical technique, omitting patient-level data, or failing to specify the acronym of the PC used, as well as those incorporating additional other ocular pathologies.

If the title or abstract did not provide adequate information, a comprehensive review of the full text was conducted to assess compliance with the inclusion criteria.

This comprehensive review encompassed fifty articles. Initially, the studies identified through database searches were 86. After removing duplicates, this number reduced to 54. Further exclusions based on title, abstract, or language criteria led to the elimination of 2 articles. After full-text review, 2 more were excluded for not meeting the inclusion criteria. Ultimately, the review included a total of 50 articles that met all the specified inclusion criteria.

In Table 1, and the accompanying pie charts 1 and 2, we summarized the number of articles considered in this review, presenting both absolute values and/or percentages, categorized by the type of PC used and the etiology of MH.

**Table 1:** n: number; Pure Platelet-Rich Plasma (PRP); Pure Platelet-Rich Plasma (PRP) Gel; Pure Platelet-Rich Fibrin (PRF); MH: Macular Hole; iFTMH: idiopathic FTMH; LMH: lamellar macular hole

MH Etiology	Number of studies per PCs forms and Macular Hole etiology				
	PRP	PRP Gel	PRF	Not specified	*n* total (%)
iFTMH	20	2	1	0	23 (46)
Refractory FTMH	11	3	1	0	15 (30)
Recurrent FTMH	1	0	0	0	1 (2)
Secondary FTMH	6	2	0	0	8 (16)
LMH	2	0	0	1	3 (6)
*n* total (%)	40 (80)	7 (14)	2 (4)	1 (2)	50 (100)

**Pie chart 1:**
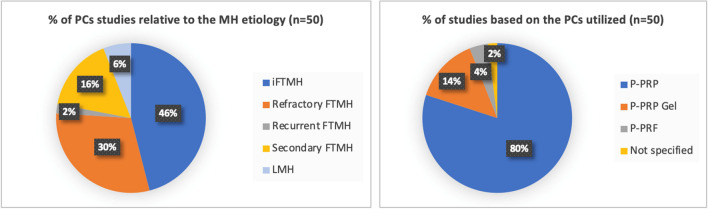
Depicting the percentage (%) of studies on platelet concentrates (PCs in the treatment of macular holes, categorized by the etiology of the macular holes and the type of platelet concentrate used. MH: Macular Hole; P-PRP: Pure Platelet-Rich Plasma; P-PRP Gel: Pure Platelet-Rich Plasma Gel; P-PRF: Pure Platelet-Rich Fibrin; iFTMH: idiopathic FTMH; LMH: lamellar macular hole

**Pie chart 2:**
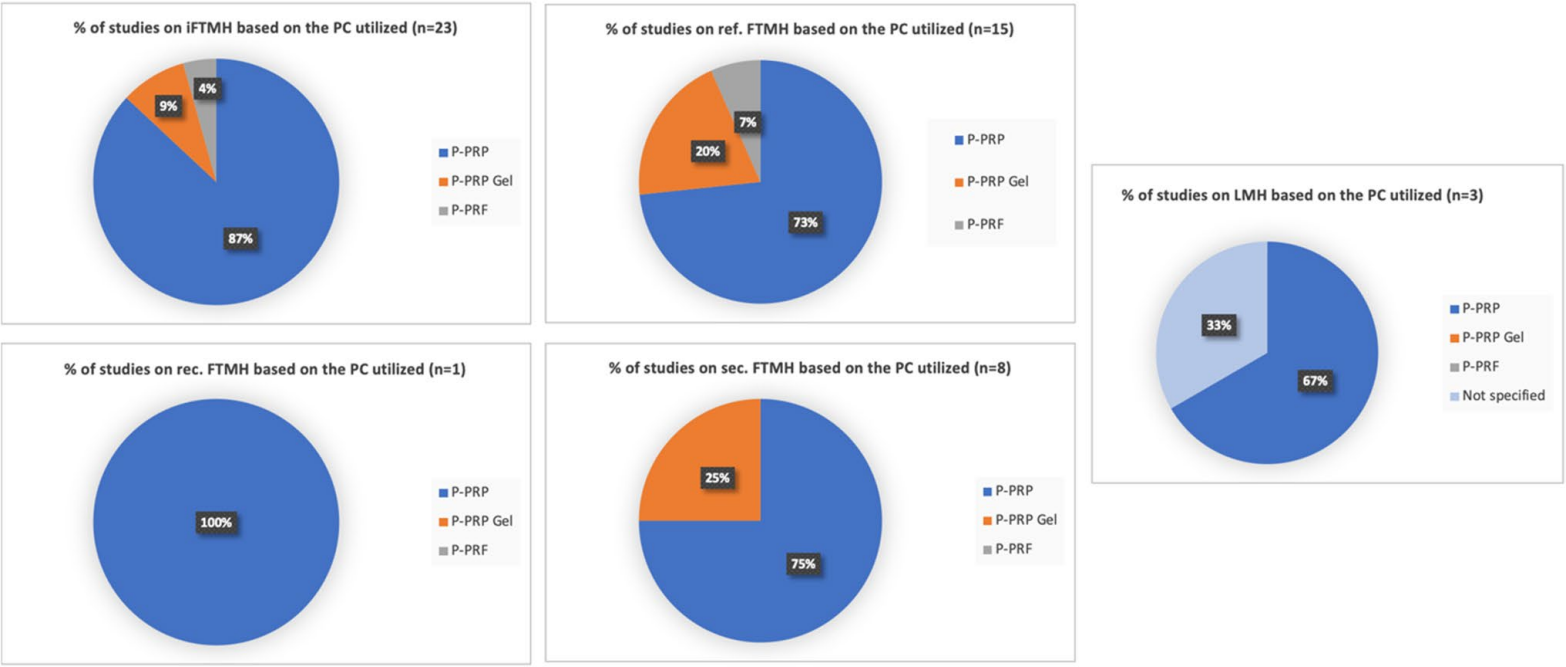
Representing the percentage (%) of studies based on the type of platelet concentrate (PC) used and the etiology of macular holes. P-PRP: Pure Platelet-Rich Plasma; P-PRP Gel: Pure Platelet-Rich Plasma Gel; P-PRF: Pure Platelet-Rich Fibrin; iFTMH: idiopathic FTMH; ref. FTMH: refractory FTMH; rec. FTMH: recurrent FTMH; sec. FTMH: secondary FTMH; LMH: lamellar macular hole

Out of 50 articles pertaining to MHs, PRP was employed 40 times (80%), PRP Gel 7 times (14%), and PRF only 2 times (4%). In one study, the specific type of PC used was not specified. The application of PCs was predominantly directed towards iFTMH at 46%, followed by refractory FTMH at 30%, secondary FTMH at 16%, LMH at 6%, and recurrent FTMH at 2%. Furthermore, considering individual etiologies, among the total studies on iFTMH, n=23, PRP was employed in 20 (87%) articles, PRP Gel in 2 (9%) articles, and PRF in 1 (4%) articles. In refractory FTMH, 11 out of 15 studies (73%) utilized PRP, 3 out of 15 (20%) employed PRP Gel, and 1 out of 15 used PRF (7%). In recurrent FTMH, a single study (100%) exclusively utilized PRP. For secondary FTMH, 6 out of 8 studies (75%) employed PRP, and in 2 out of 8 (25%), PRP Gel was used. Finally, in LMH, 2 out of 3 studies (67%) utilized PRP, while in 1 study (33%), the specific type fo PC was not specified. PRF, to date, has not been utilized in recurrent, secondary, and LMH cases.

### Data collection

The following data were collected: authors' names, year of publication, number of eyes, type of macular hole, iFTMH stage, acronym of PC used, PC classification, PCs centrifugation method, platelet concentration obtained after centrifugation, type of surgery, injected PC quantity, type of tamponade used, and postoperative positioning. All these parameters were reported in Tables 4, 5, 6, 7, and 8. If any of these parameters were not identified in the examined articles, it was recorded in the tables as “not specified.”

### Platelets concentrates preparation

**Fig. 1:**
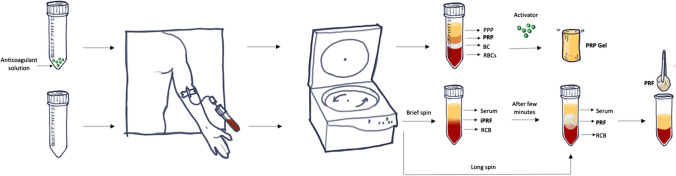
PRP preparation. PPP: platelet poor plasma; PRP: Platelet-Rich Plasma; BC: buffy coat; RBCs: red blood cells; iPRF: injectable platelet rich in fibrin; RCB: red corpuscules base; PRF: Platelet-Rich Fibrin (fibrin clot)

On the day of the surgical procedure, a peripheral blood sample is collected from the patient and placed in a test tube. The test tube may or may not contain an anticoagulant solution, typically CPDA (citrate phosphate dextrose adenine), ACD (acid citrate dextrose) or sodium citrate. Subsequently, the test tube is subjected to centrifugation to separate the various blood components and to obtain the platelet concentrate. Until now, there is no standardized centrifugation procedure in terms of the number of cycles or the speed range (revolutions per minute-rpm) used. If the centrifugation is performed on the test tube containing whole blood without an anticoagulant, the process will result in the formation of a clot containing platelets, known as the platelet-rich fibrin (PRF). On the contrary, if the centrifugation is carried out on the test tube containing whole blood and an anticoagulant, the result will be the liquid form of PRP. The liquid PRP can further be activated into the PRP gel through the addition of an activator such as calcium chloride, calcium gluconate, thrombin, batroxobin or others (Fig. 1).

It is important to note that the PRP gel differs from PRF in that their coagulation formation processes are distinct. The PRP gel requires an activator for clot formation to neutralize the anticoagulant, while the solid PRF forms through the spontaneous coagulation cascade. The biological, preparation, and concentration differences among PRP, PRP Gel, and PRF are summarized in Table 2.

**Table 2:** Characteristics of pure platelet-rich plasma (PRP), pure platelet-rich plasma (PRP) gel, and pure platelet-rich fibrin (PRF)

	PRP	PRP gel	PRF
Test tubes with Anticoagulant	Yes	Yes	No
Activators	No	Yes	No
Amount of fibrin	Absent	Low	High
Fibrin architecture	Absent	Tetramolecular (weak polymers of fibrin)	Trimolecular (strong three-dimensional matrix)
Growth factors release time	Fast after tissue contact	Fast after activation	Slow and longer
Amount of released growth factors	Good	Good	High
Matrix proteins release (Thrombospondin, Fibronectin, Vitronectin)	Fast after tissue contact	Fast after activation	Slow and longer
Technique of production	Time consuming and more expensive	Time consuming and more expensive	Less time consuming and less expensive
Final contents of platelets after preparation	Variable (based on the initial blood collection, procedure of preparation, and operator-dependent)	Variable (based on the initial blood collection, procedure of preparation, and operator-dependent)	Large

### Platelet concentrates terminology

To date, the terminology concerning platelet concentrates used in studies related to MHs has been highly dissimilar, including different terms like “platelet-rich plasma (PRP),” “platelet-rich fibrin (PRF),” “autologous platelets,” “autologous adjuvants,” “plasma rich in growth factors (PRGF),” and “platelet concentrate (PC)”. Furthermore, the acronym “APC” has been used to define four different terms: “autologous platelet concentrate” [36], “autologous thrombocyte serum concentrate” [37], “autologous Plasma Concentrate” [38] and “autologous conditioned plasma” [38], leading to terminological confusion. In reality, according to a consensus terminology [9], only four categories of platelet concentrates are to be considered: P-PRP, L-PRP, P-PRF, and L-PRF. In vitreoretinal surgery, due to the vitreal immunological sanctuary and their limited efficacy, forms containing leukocytes are no longer used. Therefore, the forms employed in ophthalmology are exclusively three: P-PRP, P-PRP gel and P-PRF. Since all three components are 'pure,' it is possible to simplify the acronym to PRP, PRP Gel and PRF. Among all the PCs definitions found in the literature, only one fall into the solid PRF group, called by the authors “platelet-rich fibrin (PRF)” [39], while all the others belong to the PRP/PRP gel category (Table 3). It is important to note that the term “plasma rich in growth factors” (PRGF) always refers to PRP/PRP gel, which, being concentrated, increases consequently the growth factors it contains.

**Table 3:** Platelet concentrates terminology used in ophthalmology

Pure Platelet-Rich Plasma (PRP or PRP Gel)	Pure Platelet-Rich Fibrin (PRF)
Platelet-Rich Plasma (PRP)	Platelet-Rich Fibrin (PRF)
Autologous platelets	
Autologous plasma	
Autologous adjuvants	
Plasma rich in growth factors (PRGF)	
Platelet concentrate (PC)	
Autologous platelet concentrate (APC)	
Autologous thrombocyte serum concentrate (APC)	
Autologous conditioned plasma (APC)	
Autologous Plasma Concentrate (APC)	

### Platelet concentrates during surgery

Three trocars are inserted via the pars plana. A central vitrectomy is performed, extending to the mid-periphery, with peeling of the ERM/ILM in primary MH. During secondary surgery, consider extending the ERM/ILM peeling. Perform a fluid-air exchange. Subsequently, the procedure varies depending on the type of PC used:

- PRP: using a 25-30 gauge needle, 3-4 drops of P-PRP are instilled inside the MH
- PRP Gel: an activator is imperative for the transformation of PRP into PRP gel, as described earlier. There exist three distinct methods for obtaining PRP gel: i) An activator is inserted into the test tube containing PRP just before its injection. It takes approximately 30–60 s for the liquid PRP to transition into a gel. During this time interval, employ a 25-gauge needle to introduce 3–4 drops of liquid PRP into the MH, inducing its transformation into a gel within the aperture. If gelation occurs within the vitreous cavity, manipulation with forceps is feasible, followed by insertion into the MH, facilitated by PFCL or cohesive viscoelastic. Excess gel can be reshaped using a vitrectomy cutters; ii) following the instillation of 3–4 drops of PRP into the MH using a 25 Gauge cannula, the activator is administered through a separate cannula (25-30-36 Gauge), over the MH. This procedure initiates the gel activation ‘on-site’; iii) the activator is introduced into the vial containing PRP, leading to the formation of the gel within the same container. The gel is then transferred to a shaper and compressed with a mold to produce a membrane plug. The membrane plug can be cut and rolled for ease of insertion onto the trocars, manipulated within the vitreous cavity using ILM forceps, and positioned inside the MH under perfluorocarbon liquid (PFCL) or cohesive viscoelastic
- PRF: Remove the fibrin clot from the test tube, shape it with scissors to be slightly larger than the MH. Insert 25 Gauge forceps through the first trocar, exit through the second trocar, retrieve the shaped clot, and place it inside the hole. If it proves to be excessively large, modify its size using a vitrectomy cutters in proximity to the hole, and subsequently reintroduce it.

Finally, tamponade is carried out using gas or polydimethyloxane (PDMS), and the patient is placed in a supine position for 2-12 h postoperative hours, followed by a face-down position based on the surgeon’s preference.

### Platelet concentrates and macular hole surgery

#### Platelet concentrates and lamellar macular hole

The use of platelets concentrate as a therapeutic adjuvant for the closure of LMH is still in its early stages, and it has been utilized only in a few cases. Although lamellar holes have recently been precisely defined based on etiology and clinical presentation by Hubschman et al. [40], clear treatment guidelines have not yet been established. Furthermore, while some authors have only partially suggested the use of a surgical approach, others have demonstrated much more promising results in terms of anatomical and functional recovery through surgery [41–43]. Regarding the use of PRP as an adjuvant therapy in LMHs surgery, there are still few publications available in the literature. The first research group that reported the surgical outcomes using PRP for LMHs was the group of Gonzalez and colleagues in 2019 [44]. In their case series, all patients obtained an anatomic closure of the foveal defect with a best-corrected visual acuity (BCVA) improvement.

In 2021, Hagenau and colleagues [45] published the results of their initial case series of 8 patients, reporting a restored foveal anatomy and an improved BCVA at the 3-month follow-up visit after performing a 23 PPV with ILM peeling and injection of 0.1 ml of highly concentrated PRP.

More recently, the same authors published a long-term follow-up of 19 patients treated with 23/25G PPV and 0.1ml of PRP injection. They observed a restoration of foveal anatomy in all patients and a significant improvement in BCVA (0.33 ± 0.15 logMAR preoperative and 0.18 ± 0.13 logMAR postoperative, *p* = 0.028), indicating both morphological and functional improvement at the long-term follow-up. Interestingly, they also reported a recurrent foveal defect at the 6 months follow-up for the only two patients who had not received ILM peeling. Furthermore, the BCVA improvement was confirmed despite the lens status in a subgroup analysis of the 8 pseudophakic patients. (Table 4)

**Table 4:** Studies utilizing platelet concentrates in lamellar macular holes

Author/Year	Eyes (n)	Acronymous used	Platelet concentrates classification	Centrifugation	Platelets concentration	Surgery	Tamponade	Post operative Posture	Quantity Injected (ml)
Gonzalez et al, 2019	3	Autologous platelet plug	Not Specified	Not specified	Not specified	23G-PPV	C3F8	Supine	0.1
Hagenau et al, 2021	8	Platelet rich plasma (PRP)	PRP	Closed-circuit centrifugation procedure (Arthrex Angel SystemTM; Arthrex, Naples, Florida, USA)	8.8× higher than in whole blood	23G-PPV + ILM Peeling (and ERM if present)	Air or Gas (not specified)	Supine 2 h	0.1
Hagenau et al, 2023	19	Platelet rich plasma (PRP)	PRP	Closed-circuit centrifugation procedure (Arthrex Angel SystemTM; Arthrex, Naples, Florida, USA)	8.8× higher than in whole blood	23/25G-PPV + ILM Peeling (and ERM if present)	Air or Gas (not specified)	Supine 2 h	0.1

#### Platelet concentrates and idiopathic full-thickness macular hole

The first adoption of PRP (0.03 ml) in the surgical management of iFTMHs dates back to 1995. Ligget et al. pioneered its application as an adjunctive treatment with PPV for stage III and stage IV iFTMHs and C3F8 as tamponing agent [46]. This approach was based on their recognition of the cell proliferative effect of autologous serum in vitro and in animal models [47–51]. A total of 11 eyes were treated, resulting in a 100% closure rate of MHs and an improvement of at least two lines of visual acuity on the standard Snellen chart [46]. In the same year, Alain Gaudric et al. conducted the first comparative study between vitrectomy with and without the use of PRP (0.1 ml) in the treatment of stage II, III, and IV iFTMHs [52]. A higher MH closure rate in the PRP group (19 out of 20 eyes) was observed compared to the no PRP group (13 out of 20 eyes), with similar functional outcomes between the two groups [52]. In 1996, a pilot study, led by Jean-Francois Korobelnik, was conducted on iFTMHs of stage III and IV, involving a cohort of 6 eyes. The study demonstrated an 83.3% MH closure rate (5 out of 6 eyes) over an average follow-up period of 7 months. This closure rate was associated with a significant functional improvement of 2 or more lines on the visual acuity chart [53]. In the same year, another interventional study involving the use of PRP revealed a lower percentage of MH closure rate in stage II, III, and IV iFTMHs compared to previous PRP studies (67%, 29 eyes out of 44) [54]. Nonetheless, despite this outcome, the MH closure rate was similar to that observed with other surgical techniques available at the time [28, 29]. These findings justified and sparked enthusiasm for the application of PRP in iFTMH cases.

Since then, numerous studies assessing the efficacy of PRP in stage II, III, and IV iFTMH were conducted. Some of these studies evaluated the effectiveness of PRP as an adjunctive treatment during vitrectomy with ERM peeling, when present [39, 53, 55–58].

Specifically, Alain Gaudric et al., in their analysis of 69 operated eyes, observed an anatomical success rate of 93%, with 72% of patients achieving a visual acuity of at least 20/50. These results align with the findings reported in other relevant literature [59].

Moreover, Brendan J Vote Franzco et al., reported a high MH closure rate of 95.7% (67 eyes out of 70) with an 8.5% incidence of hole reopening, on average 12.7 months after surgery, in stage II,III, IV iFTMH [60].

Other studies have compared the use of PRP with a control group in stage II,II,IV iFTMH. Gehring et al. evaluated the anatomical success by comparing PRP derived from whole blood (12 eyes) and PRP produced from plateletpheresis (7 eyes), finding no significant differences between the two groups[61]. Hans Hoerauf et al., on the other hand, compared the use of PRP (30 eyes) versus whole blood (14 eyes) and observed a higher MH closure rate in the first group (93.9%) compared to the second group (36.4%) [62]. This difference was likely due to the lower platelet concentration and the presence of leukocytes in whole blood. As a result, to date, L-PRP and L-PRF have not been utilized in macular hole treatment.

Mulhern et al., otherwise, compared the use of two different gas tamponades after PRP injection in stage II,III,IV MH: C3F8 (31 eyes) and SF6 (31 eyes) [63]. They found an anatomical success rate of 96.7% and 93.5%, respectively (*P*=1.00). It is interesting to note that although there were no statistically significant differences between the two groups, the SF6 treated group showed faster visual acuity improvement, fewer intraocular pressure spikes, and fewer cases of subcapsular cataract development at the 3-month follow-up [63].

Minihan et al. compared three groups: PRP injection and C3F8 gas tamponade (50 eyes) vs. TGF-B2 injection and SF6 gas tamponade (15 eyes) vs. only SF6 gas tamponade in stage II,III,IV MH [64]. The anatomical success occurred in 86% of eyes, but the best surgical outcomes were achieved in the PRP group, with a 96% MH closure rate (*P*=0.01). Functional results were also superior in the PRP group, where 74% of eyes improved by more than two lines of visual acuity, compared to 65% in the SF6 tamponade group and 33% in the TGF-B2 injection group [64].

Also, retrospective studies in stage II,III,IV MH were conducted to compare the ERM/ILM peeling with or without PRP injection [65–68]. Specifically, Alexander A. Shpak et al. included 214 eyes, with 152 eyes undergoing ERM/ILM peeling alone (control group) and 62 eyes undergoing both ERM/ILM peeling and liquid PRP injection. All MHs treated with PRP were closed, while 7.2% of holes in the control group remained open at the 12-month follow-up (*P*= 0.036). Additionally, the final mean BCVA was significantly better in the PRP group (*P* = 0.012) [67].

Eric Ezra et al. analyzed three groups (observation group, 61 eyes; vitrectomy group, 59 eyes; and vitrectomy plus PRP group, 65 eyes) with a longer follow-up period of 24 months. At the end of the follow-up period, 7% of iFTMHs in the observation group, 78% in the vitrectomy group, and 86.2% in the vitrectomy plus PRP group were closed. However, no significant differences were evident for any measure of visual acuity in the surgical groups [68].

Lastly, Babu et al. compared the inverted ILM flap technique (group 1: 30 eyes) vs. ILM peeling and PRP use (group 2: 30 eyes) in the treatment of stage IV iFTMHs. They achieved an anatomical success rate of 90% (*n*=27/30) in the first group and 93.3% (*n*=28/30) in the second group, with no statistically significant differences between the groups in visual acuity at the 3-month follow-up [69].

To date, only two studies using non liquid PRP in the treatment of iFTMHs were conducted [70, 71]. In 2001, Blumenkranz et al. treated 121 eyes with stage II, III, and IV iFTMHs using an autologous plasma-thrombin mixture. This mixture consisted of PRP obtained from the patients themselves and bovine thrombin (Thrombinar/Armour Pharmaceutical Co., Kankakee, IL) [71]. Specifically, one or two drops of PRP were instilled into the macular hole using a 30-gauge needle, followed by a drop of bovine thrombin, resulting in the formation of a small translucent solid clot directly over the macular hole, the PRP gel. In this context, PRP gel referred to the activated form of the PRP. At the final follow-up (mean: 10.9 months), a MH closure rate of 81% (98 eyes out of 121) and an improvement of two or more lines of visual acuity in 78% of cases (94 eyes out of 121) were observed [71].

More recently, in 2022, a retrospective interventional study conducted by Ning Yang et al. showed a 100% of MH closure rate (17 eyes out of 17) using PRF (article available upon payment) in the treatment of stage IV iFTMHs [70]. Additionally, a significant improvement in visual acuity from baseline (1.21 ± 0.33 logMAR) to six months of follow-up (0.64 ± 0.22 logMAR) (*p*<0.001) was observed, with no intraoperative or postoperative complications reported [70]. Although the literature on the use of solid PRF in the treatment of iFTMHs is limited, it appears that this form of platelet concentrate may yield favorable anatomical and functional outcomes in the management of MHs. (Table 5)

**Table 5:** Studies utilizing platelet concentrates in idiopathic full thickness macular hole

Author/Year	Eyes (n)	Acronymous used	Platelet concentrates classification	Centrifugation	Platelets concentration	Stage iFTMH	Surgery	Tamponade	Post operative posture	Quantity injected (ml)
Ligget et al. (1995)	11	Autologous serum	PRP	30,000 rpm x 15 min	Not specified	III, IV	PPV + ERM peeling if present + P-PRP	C3F8	Face down	0.03
Gaudric et al. (1995)	40	Autologous platelet concentrate	PRP	280 g x 15 min then 1000g x 10 min	10^9^/ml	II,III,IV	PPV + ERM peeling if present + P-PRP Vs Only PPV + ERM peeling if present	C2F6-Air mixture	Supine for 24 h, then face down Vs Face down	0.1
Korobelnik et al. (1995)	6	Autologous platelet concentrate	PRP	1300 rpm then 3000 rpm then another centrifugation (not specified)	400,000 to 5,900,000 mm^3^	III, IV	PPV + ILM peeling + P-PRP	C2F6	Face down	0.03-0.04
Wells et al. (1996)	44	Autologous serum	PRP	4000 rpm x 5 min	Not specified	II,III,IV	PPV + P-PRP	C3F8/SF6	Supine until awake then face down	Not specified
Paques et al. (1997)	110	Autologous Platelet Concentrate	PRP	150 g x 15 min then 1500 g x 10 min	Not specified	III, IV	PPV + ERM peeling if present + P-PRP vs PPV + ERM peeling if present	C3F8	Supine position for 12 h the face down vs Face down	0.1
Minihan et al. (1997)	85	Autologous platelet concentrate	PRP	280 gr x 15 min Then 1000 gr x 10 min	Not specified	II, III, IV	PPV + P-PRP Vs PPV Vs PPV + TGF- β 2	C3F8/SF6	Supine for 6 h then face down	0.1
Gaudric et al.(1997)	69	Platelet Concentrate	PRP	150 gr x 15 min Then 1500 gr x 10 min	Not specified	II,III,IV	PPV + ERM peeling if present + P-PRP	C2F6 and Air	Supine for 12 h then face down	0.1
Gehring et al. (1999)	19	Autologous platelets	PRP	200 gr x 20 min Then 200 gr x 15 min	10 x10^9^ ml	II,III,IV	PPV + P-PRP Vs PPV + whole blood	C2F6	Supine for 3 h then face down	<0.2
Banker et al. (1999)	164	Autologous serum	PRP	350 gr x 10-15 min	Not specified	III,IV	PPV + ERM peeling + P-PRP Vs PPV + ERM peeling	C3F8	Face down	0.06
Mulhern et al. (2000)	62	Autologous platelet concentrate	PRP	280 gr x 15 Then 1000 gr x 10	Not specified	II,III,IV	PPV + ERM peeling if visibile + P-PRP	C3F8/SF6	Supine for 4 h then face down	0.1
Blumenkranz et al. (2001)	121	Plasma-Thrombin mixture	PRP gel	1000 g x 10 min + bovine thrombin	Not specified	II,III,IV	PPV + ERM peeling if visibile + P-PRP gel	C2F6/C3F8	Prone	0.01-0-02
Hoerauf et al. (2002)	40	Platelet concentrate	PRP	200g x 20 min Then 2000 x 15 min	Not specified	II,III,IV	PPV + P-PRP Vs PPV + whole blood	C2F6	Supine for 3 h then face down	0.1
Franzco et al. (2004)	70	Autologous platelets	PRP	150 gr x 15 min Then 1500 x 10 min	Not specified	II, III, IV	PPV + P-PRP	C3F8	Supine overnight then face down	0.1
Ezra et al. (2004)	185	Autologous serum	PRP	3000 x 10 min	Not specified	II, III, IV	PPV + ERM peeling + P-PRP Vs PPV + ERM peeling Vs Only observation	C3F8	Face down	0.25
Cheung et al. (2005)	56	Aautologous platelets	PRP	Not specified	Not specified	II,III,IV	PPV + P-PRP	C3F8/SF6	Face down	Not specified
Kapoor et al (2012)	13	Autologous platelets	PRP	Not specified	Not specified	Not specified	PPV + ILM peeling + P-PRP	C3F8	No positioning	Not specified
Konstantinidis et al. (2013)	21	Autologous platelets	PRP	280 g (1500) x 15 min Then 1000 g x 10 min	Not specified	III,III,IV	23 G PPV + P-PRP	C3F8	Face down	0.1
Kung et al. (2013)	38	Autologous serum	PRP	3000 rpm x 10	Not specified	III, III, IV	20-23 G PPV + ILM peeling + P-PRP Vs 20-23 G PPV + ILM peeling	C3F8	Face down	0.2
Babu et al. (2020)	60	Platelet rich plasma	PRP	REMI PRP centrifuge machine (REMI Group, India). 1^st^ centrifugation: 15 min 2^nd^centrifugation: 6 min	Not specified	IV	25 G PPV + ILM peeling + P-PRP Vs 25 G PPV + inverted flap	SF6	Supine Vs Face down	Not specified
Shpak et al. (2021)	214	Autologous platelet-rich plasma	PRP	4000 x 5 min	10^7^-10^9^/ml	II,III.IV	25-27 G PPV + ILM peeling + P-PRP Vs 25-27 G PPV + ILM peeling	Air	Face down position	Not specified
Kim et al. (2021)	Not specified	Autologous Platelet Concentrate	PRP	3000 x 3 min	Not specified	IV	25 G PPV + ILM peeling + P-PRP Vs 25 G PPV + ILM peeling +/- ILM flap tecnique	C3F8	Supine for 6 h then face down Vs Face down	0.1
Sánchez-Ávila et al. (2022)	2	Plasma rich in growth factors membrane	PRP gel	Endoret closed system (PRGF, Ophthalmology kit, BTI Biotechnology Institute, S.L., Miñano, Álava, Spain): 580 g x 8 min + 10% CaCl (Endoret Activator (BTI Biotechnology Institute, S.L., Miñano, Álava, Spain)	Not specified	IV	23 G PPV + ILM peeling + P-PRP gel	C3F8	Face down for 3 days then supine	Not specified
Yang et al. (2022)	17	Platelet rich in fibrin	PRF	3000 rpm x 10 min (Scilogex, DMO421, Rocky Hill, CT, USA)	Not specified	IV	23 G PPV + ILM peeling + P-PRF	Air	Face down for 7 days	clot of the size of the macular hole

#### Platelet concentrates and persistent/refractory FTHM

The use of PRP as an adjunctive treatment in vitreoretinal surgery for persistent/refractory MHs dates back to 1996, when Korobelnik et al. achieved anatomical success in two cases of stage 4 MH. These MHs had previously failed to heal following vitrectomy and gas tamponade [53].

Two years later, in 1997, Gaudric et al. attempted to assess the effectiveness of this compound not only in iFTMH but also in 8 MHs that had previously failed to close after the initial surgery without the use of platelet compound [59]. The overall anatomical success rate was 93% (72 eyes out of 77), without specifying whether the non-closed holes were iFTMHs or persistent/refractory ones [59].

Subsequently, the use of PRP in the treatment of this type of MHs was only resumed in 2015, when Figueroa et al. demonstrated a complete anatomical success at 6 months of follow-up in 2 cases of myopic MHs refractory to initial surgery [72]. It is interesting to note that, for the first time in recent times, this could be considered an alternative approach to the inverted ILM flap technique with comparable anatomical and functional outcomes.

From that moment, a modest number of articles regarding the use of PRP in the treatment of persistent/refractory MHs were published [37, 56, 57, 73–77] [78]. Specifically, Degenhardt et al. employed PRP in 103 eyes with persistent MHs (size from 292 to 529 μm) following vitrectomy with ILM peeling and gas tamponade [79]. They observed a MH closure rate of 60.2% (62 eyes out of 103) at a median follow-up of 60 days. Through a multivariate analysis, the authors identified tractional hole index, axial length, time between the first and second surgery, and surgeon experience as predictive factors for MH closure [79].

Purtskhvanidze and colleagues also evaluated the anatomical and functional outcomes of using PRP in persistent FTMH and compared them with the use of autologous whole blood [80]. 61 eyes underwent treatment with PRP (group 1), while 14 eyes received autologous whole blood (group 2). The MH diameter before revitrectomy was 446 ± 155 μm. Indeed, the results are intriguing. Among the patients in group 1, 82.2% (52 out of 61) achieved anatomical success, whereas in group 2, only 7.1% (1 out of 14) experienced success over an average follow-up period of 58 months [80].

For the first time, even in persistent MHs, a low MH closure rate with the use of autologous whole blood was observed, suggesting a possible negative or worthless effect of leukocytes on glial proliferation.

Another retrospective study conducted by Schaub et al. compared the use of PRP and gas tamponade, SF6, (13 eyes) versus the application of only heavy silicon oil (Densiron® 68) (35 eyes) in persistent MHs (446 ± 155 μm of minimum linear diameter) [81]. The MH closure rate in the PRP group was 57.1%, while it was 45.7% in the heavy silicon oil group, and this difference was not statistically significant (*p*=0.102). Additionally, when BCVA at 2 months post-surgery in the PRP group with BCVA at 2 months after silicone oil removal in the heavy silicon oil group, no significant functional differences were observed (*p* ⩾ 0.741) [81].

However, a significantly better functional outcome (*p*=0.019) was observed in non-closed MHs following a second vitrectomy, where the application of PRP was utilized. Considering the non-inferiority of PRP, the inflammatory properties of silicone oil, and the need for a second re-intervention for its removal, the authors recommended the use of platelet compounds in the treatment of persistent MHs [81].

Finally, the sole case report examining the application of PRF in refractory MHs was conducted by Arif Koytak et al. in 2019 [82]. Specifically, they treated two refractory MHs using PRF and SF6 gas tamponade, leading to their closure at a 3-month follow-up. Furthermore, the BCVA in both eyes showed improvement, transitioning from counting fingers to 0.16 LogMAR in the first case and from 0.05 to 0.02 LogMAR in the second case, both at the 3-month follow-up. No intraoperative or postoperative complications were reported [82].

In this context, it is essential to emphasize that the PRF denotes the coagulated form of whole blood. After collecting the patient's whole blood, centrifugation was performed in a tube without anticoagulant factors. The absence of anticoagulant factors facilitated blood coagulation. Subsequently, the fibrin clot was extracted from the tube, compacted with a metal press board, and utilized to plug the retinal hole [82] (Table 6).

**Table 6:** Studies utilizing platelet concentrates in persistent/refractory full thickness macular hole

Author/Year	Eyes (*n*)	Acronymous used	Platelet concentrates classification	Centrifugation	Platelets concentration	Surgery	Tamponade	Post operative posture	Quantity injected (ml)
Korobelnik et al. (1995)	2	Autologous platelet concentrate	PRP	1300 rpm then 3000 rpm then another centrifugation (not specified)	400,000 to 5,900,000 mm^3^	PPV + ILM peeling + P-PRP	C2F6	Face down	0.03-0.04
Gaudric et al.(1997)	8	Platelet Concentrate	PRP	150 gr x 15 min Then 1500 gr x 10 min	Not specified	PPV + ERM peeling if present + P-PRP	C2F6 and Air	Supine for 12 h then face down	0.1
Figueroa et al. (2015)	2	Platelet rich plasma	PRP	1600 x 10 min	Not specified	23 G PPV + ILM peeling + P-PRP	C3F8/PDMS	Supine for 30 min then face down	Not specified
Dimopoulos et al. (2017)	27	Autologous thrombocyte serum concentrate	PRP	Not specified	Not specified	PPV + P-PRP	Gas	Not specified	Not specified
Purtskhvanidze et al. (2017)	75	Autologous Platelet Concentrate	PRP	200g x 20 Then 200g x 15 min	10 × 10^9^ platelets/mL	20-23 G PPV + P-PRP Vs 20-23 G PPV + whole blood	C2F6	Supine for 12 h then face down	Not specified
Bringmann et al. (2019)	8	Autologous platelet concentrate	PRP	500 g x 10 min then 500 g x 10 min	1-5×10^10^/mL	20-23-25 G PPV + addition ILM peeling + P-PRP	C3F8/SF6	Supine for 2-24 h then face down	Not specified
Degenhardt et al. (2019)	103	Autologous platelet concentrate	PRP	500 g x 10 min then 500 g x 10 min	1-5×10^10^/mL	20-23-25 G PPV + addition ILM peeling + P-PRP	C3F8/SF6	Supine for 12 h then face down	Not specified
Arias et al. (2019)	2	Platelet rich in growth factors	PRP gel	Endoret protocol: 580 g x 8 min + 10% CaCl2 (PRGF–Endoret activa- tor; BTI Biotechnology Institute, Vitoria, Spain)	Not specified	23-25 G PPV + P-PRP gel	PDMS	Not specified	Not specified
Koytak et al. (2019)	2	Platelet-Rich Fibrin	PRF	Not specified	Not specified	PPV + P-PRF	SF6	Face down	Not specified
Schaub et al. (2020)	48	Autologous platelet concentrate	PRP	Not specified	10 × 109 platelets/mL	20-23 G PPV + addition ILM peeling + P-PRP Vs 20-23 G PPV + addition ILM peeling	SF6 Vs Densiron	Supine for 24 h then face down Vs none	Not specified
Figueroa et al. (2020)	11	Platelet rich in growth factors	PRP gel	Endoret closed system (PRGF, Ophthalmology kit, BTI Biotechnology Institute, S.L., Miñano, Álava, Spain): 580 g x 8 min + 10% CaCl (Endoret Activator (BTI Biotechnology Institute, S.L., Miñano, Álava, Spain	Not specified	23 G PPV + addition ILM peeling + P-PRP gel	C3F8	Not specified	Not specified
Kim et al. (2021)	Not specified	Autologous Platelet Concentrate	PRP	3000 x 3 min	Not specified	25 G PPV + ILM peeling + P-PRP Vs 25 G PPV + ILM peeling +/- ILM flap tecnique	C3F8	Supine for 6 h then face down Vs Face down	0.1
D’Alterio et al. (2022)	1	Platelet-rich plasma	PRP	200 x 10 min	Not specified	25 G PPV + P-PRP	C2F6	Supine for 2 h then face down	0.1
Sánchez-Ávila et al. (2022)	2	Plasma rich in growth factors membrane	PRP gel	Endoret closed system (PRGF, Ophthalmology kit, BTI Biotechnology Institute, S.L., Miñano, Álava, Spain): 580 g x 8 min + 10% CaCl (Endoret Activator (BTI Biotechnology Institute, S.L., Miñano, Álava, Spain)	Not specified	23G PPV + addition ILM peeling + P-PRP gel	C3F8/PDMS	Face down for 3 days then supine	Not specified
Buzzi et al. (2023)	28	Autologous Platelet-Rich Plasma	PRP	1600 x 10 min	Not specified	PPV + P-PRP	C2F6	Supine for 1 h then face down	Not specified

#### Platelet concentrates and recurrent FTMH

To date, the only prospective, interventional, multicenter study found in the literature that used a PC of PRP for the treatment of recurrent MHs is the one conducted by Kim et al. in 2021 [76]. Specifically, they enrolled 117 eyes with recurrent MHs, large MHs, or MHs with high myopia (minimum diameter from 510 to 618 μm). Out of these, 59 eyes underwent PPV with ILM remnant peeling +/- ILM flap technique (subject to the surgeon's discretion) and gas tamponade (control group), while 58 eyes underwent PPV with ILM remnant peeling, PRP injection, and gas tamponade (experimental group). At a 6-month follow-up, MH closure was achieved in 79.7% of the control group and in 89.7% of the experimental group, with no significant differences between the two groups (*p* = 0.134). The subgroup analysis demonstrated anatomic success in recurrent MHs of 57.1% in the control group and 60.0% in the experimental group (*p* = 0.921), without specifying the number or size of recurrent FTMHs. Although both BCVA and metamorphopsia, measured using the M-chart score, improved over time in both groups, no significant differences were observed between the groups during the follow-up period (BCVA, *p* = 0.130; M-chart score, *p* = 0.762) [76]. Consequently, for the authors the additional use of P-PRP for recurrent MHs proved non-inferior to conventional MH surgery (Table 7).

**Table 7:** Studies utilizing platelet concentrates in recurrent full thickness macular hole

Author/Year	Eyes (n)	Acronymous used	Platelet concentrates classification	Centrifugation	Platelets concentration	Surgery	Tamponade	Post operative posture	Quantity Injected (ml)
Kim et al. (2021)	Not specified	Autologous Platelet Concentrate	P-PRP	3000 x 3 min	Not specified	25 G PPV + ILM peeling + P-PRP Vs 25 G PPV + ILM peeling +/- ILM flap tecnique	C3F8	Supine for 6 h then face down Vs Face down	0.1

#### Platelet concentrates and secondary FTHM

For the first time, the use of PRP in secondary myopic FTMH can be dated to 2001, when Hoerauf et al. enrolled 44 eyes, comprising 40 eyes with iFTMHs and 4 eyes with myopic MHs [62]. The results of the study have already been mentioned in the ‘Platelets concentrates and iFTMH’ section. The study did not provide a subgroup analysis for secondary MH [62].

Subsequently, Figueroa et al. reported the short-term results of PRP treatment for high myopic MH in 2016 [72], followed by the long-term results with PRP gel in 2020 [56]. The latter study was a monocentric, single-surgeon, retrospective study that enrolled both naïve high myopic MH (31 eyes, Group 1) and persistent high myopic MH (11 eyes, group 2) without specifying the minimum linear diameter. Thereafter the surgical procedure, anatomical success was achieved in 90% (28/31 eyes) of group 1 and 91% (10/11 eyes) of group 2, with a minimum follow-up period of 12 months. Interestingly, positive functional predictive factors before surgery were the presence of intraretinal cysts (*p*=0.028) and elevated FTMH borders (*p*=0.005), while a negative functional predictive factor was the dome-shaped macula (*p*=0.049) [56]. For all eyes, a commercial ready-to-use PRP gel system (Endoret® kit, BTI Biotechnology Institute, S.L., Miñano, Álava, Spain) was utilized. Peripheral patient blood was collected and subjected to centrifugation using the commercial kit. The PRP obtained was denoted by the authors as “plasma rich in growth factors” (PRGF). During the surgical procedure, the PRP was blended, and then activated in PRP-gel, with calcium chloride before being instilled into the macular holes [56].

Finally, in the aforementioned study, Kim et al. [76], through a subgroup analysis, revealed an anatomical success rate of 84.6% in the control group (PPV) and 94.7% in the experimental group (PPV + PRP) concerning myopic MH treatment. Nevertheless, this difference did not achieve statistical significance (*p* = 0.146) [76].

Additionally, case reports on the utilization of PRP or PRP gel in the treatment of MH secondary to macular telangiectasia type 2 [83] and trauma [36, 84] were published. Delving deeper, Finn et al., in 2021, described a novel technique in the context of platelet preparations: the ILM flap technique plus PRP and C3F8 tamponade for the treatment of a large (1390 μm) traumatic MH in a pediatric patient [38]. At a 3-month follow-up the hole was closed. The authors suggested this technique as a surgical option for large MHs, as it encompassed both the benefits of the ILM flap (acting as a strong plug and scaffold) and those of using PRP (facilitating the release of growth factors and cellular adhesion) [38] (Table 8).

**Table 8:** Studies utilizing platelet concentrates in secondary full thickness macular hole

Author/Year	Eyes (n)	Type of secondary FTMH	Acronymous used	Platelet concentrates classification	Centrifugation	Platelets concentration	Surgery	Tamponade	Post operative Posture	Quantity Injected (ml)
Hoerauf et al. (2002)	4	Myopic	Platelet concentrate	PRP	200g x 20 min Then 2000 x 15 min	Not specified	PPV + P-PRP Vs PPV + whole blood	C2F6	Supine for 3 h then face down	0.1
Wachtlin et al. (2003)	4	Traumatic	Platelet Concentrate	PRP	Not specified	4x10^9^/ml	PPV + ILM peelinh + P-PRP	SF6	Face down	Not specified
Figueroa et al. (2015)	7	Myopic	Platelet rich plasma	PRP	1600 x 10 min	Not specified	23 G PPV + ILM peeling + P-PRP	C3F8/PDMS	Supine for 30 min then face down	Not specified
Coca et al. (2017)	1	Traumatic	Autologous platelet concentrate	PRP	200 g x 20 min then 2000 x 15 min	Not specified	23 G PPV + ILM peeling + P-PRP	SF6	Not specified	Not specified
Rangel et al. (2020)	1	Macular telangiectasia type 2	Plasma rich in growth factors membrane	PRP gel	Endoret closed system (PRGF, Ophthalmology kit, BTI Biotechnology Institute, S.L., Miñano, Álava, Spain): 580 g x 8 min + 10% CaCl (Endoret Activator (BTI Biotechnology Institute, S.L., Miñano, Álava, Spain)	Not specified	PPV + ILM peeling + P-PRP gel	PDMS	None	Not specified
Figueroa et al. (2020)	42	Myopic	Platelet rich in growth factors	PRP gel	Endoret closed system (PRGF, Ophthalmology kit, BTI Biotechnology Institute, S.L., Miñano, Álava, Spain): 580 g x 8 min + 10% CaCl (Endoret Activator (BTI Biotechnology Institute, S.L., Miñano, Álava, Spain	Not specified	23 G PPV + ILM peeling + P-PRP gel	C3F8	Not specified	Not specified
Kim et al. (2021)	Not specified	Myopic	Autologous Platelet Concentrate	PRP	3000 x 3 min	Not specified	25 G PPV + ILM peeling + P-PRP Vs 25 G PPV + ILM peeling +/- ILM flap tecnique	C3F8	Supine for 6 h then face down Vs Face down	0.1
Finn et al. (2021)	1	Traumatic	Autologous conditioned plasma	PRP	Arthrex APC kit (Arthrex Inc, Naples, FL): 1500 x 5 min	Not specified	25 G PPV + ILM flap technique + P-PRP	C3F8	Face down	Not specified

#### Expert opinion

FTMH and LMH can lead to a decline in central visual acuity and quality of life if left untreated. To determine the most suitable surgical technique for achieving a high likelihood of closure, it is essential to classify the retinal defect.

In 2005, the initial classification system for FTMH categorized them into four stages: Stage 1 (impending macular hole), Stage 2 (≤ 250 μm or > 250 to ≤ 400 μm with vitreomacular traction), Stage 3 (> 400 μm with vitreomacular traction), and Stage 4 (FTMH with posterior vitreous detachment)[85]. Subsequently, in 2013, the International Vitreomacular Traction Study Group (IVTS) further classified macular holes based on the minimal linear diameter (MLD) in micrometers (μm), defining them as small (≤250 μm), medium (>250 to ≤400 μm), and large (> 400 μm) [4].

Based on these diameters, closure rates approaching 100% have been achieved for small and medium MH subjected to PPV with ILM peeling and gas tamponade, while large MHs exhibited closure rates of approximately 80% [86]. This underscores that MHs larger than 400 microns do not respond as favorably to conventional surgery compared to smaller holes. Furthermore, an additional classification for large MHs became necessary, recognizing that a 400 μm MH cannot be equated with a 1000 μm MH, despite both being categorized as large [21, 87]. Therefore, in 2023, the Close Study Group proposed a new classification based on the MLD: < 250 μm as Small, >250 μm to ≤400 μm as Medium, >400 μm to ≤550 μm as Large, >550 μm to ≤800 μm as X-Large, >800 μm to ≤1000 μm as XX-Large, >1000 μm as Giant [88].

The study group demonstrated that conventional ILM peeling had a MH closure rate of 97% in large MH, decreasing to 80% in XX-large MH. In contrast, newer additional techniques, such as the hAM patch and ILM flap technique, ensure a high MH closure rate with less dependence on the MLD. Therefore, ILM peeling remains the surgical gold standard up to large MHs, while X-large, XX-large, and Giant MHs can be successfully treated with additional surgical maneuvers, including ILM flap techniques, hAM, macular hydrodissection, and autologous retinal transplantation [88].

The use of PCs (PRP, PRP Gel, and PRF) is emerging as an alternative option in the surgical landscape of MHs, providing an alternative to additional surgical techniques. Indeed, when compared to the conventional surgical technique (ILM peeling alone) in stage II, III, IV MHs, regardless of their etiology, the supplemental use of PRP has yielded disparate outcomes. In the majority of scientific studies, no statistically significant differences in MH closure rates have been observed [52, 65, 66, 68, 76, 89], while a solitary study demonstrated a higher MH closure rate (*P*=0.036) [67]. No study has directly compared the additional use of PCs with other recent surgical techniques, such as hAM patch, macular hydrodissection, and autologous retinal transplantation, and there has been no comparison among different PCs.

Regarding the use of PRP Gel based on the size of the MH, it has been employed in the treatment of 121 stage II, III, IV iFTMH, achieving a MH closure rate of 81% [71]. In two iFTMH with MLD measuring 499 μm and 547 μm, a 50% MH closure rate was observed [57]. PRP Gel has also been utilized in 15 refractory medium and large MHs, resulting in closure rates ranging from 91 to 100%, and in small, medium, large, and X-large secondary MH, obtaining a 90% MH closure rate [56, 57, 75].

Furthermore, the application of PRF in MHs has been described only twice: in 17 large, X-large, and XX-large MHs [70], and in 2 Giant MHs, achieving a 100% surgical success rate [82].

To date, no differences MH closure rates have been observed when using PCs and C3F8 or SF6 or PDMS as tamponading agents [55, 71, 72, 75, 77, 79, 81]. Moreover, in all studies, no adverse events related to the use of PCs have been reported.

In light of these findings, it would be beneficial the use of PCs for MHs with a MLD greater than 550 μm (X-Large, XX-Large, and Giant). In X-large MHs, this surgical technique would stand as an alternative to ILM flap techniques, while in XX-large and Giant holes, it would offer an alternative to hAM, macular hydrodissection, and autologous retinal transplantation techniques. However, considering its faster, less invasive nature on the retinal surface, easier execution, requiring less surgical training, and yielding comparable postoperative outcomes to the aforementioned techniques, it could be suggested as a first-line option.

From the perspective of choosing between PRP, PRP Gel, and PRF in terms of preparation, it would be advisable to use the first two, depending on the surgeon's preference and experience, surgical training, and the availability of the preparation in the working facility. PRF, in fact, has limited literature evidence (2 studies) involving a small number of eyes (19), although this product, due to its high amount and prolonged release of growth factors and a much stronger fibrin architecture, may prove beneficial in XX-large and Giant MHs.

However, specific instances may warrant a preference for one PC over the other:

- LMH: advocating for the use of PRP is based on its more effective filling of intraretinal delamination compared to PRP Gel
- Less experienced vitreoretinal surgeon: recommending the use of PRP is based on its involvement in a simpler surgical technique compared to PRP Gel
- Non-compliant patient with postoperative positioning: suggesting the use of PRP Gel is based on its denser preparation, which is less likely to dislocate in the early postoperative hours, even in the absence of specific head postures
- High myopic MH with staphyloma: recommending the use of PRP Gel is based on the aim to uniformly fill the MH and avoid potential partial filling by liquid PRP at the most dependent point of the hole.

Ultimately, as a tamponade agent, it could be recommended the utilization of gas, with a preference for SF6 over C3F8 and PDMS. SF6, in particular, preserves the MH closure rate, exhibiting a diminished incidence of intraocular pressure spikes, reduced onset of subcapsular cataracts, and accelerated postoperative visual acuity recovery, obviating the need for a secondary intervention for its removal.

## Conclusion

The management of FTMH and LMH in ophthalmology has seen continuous development, with a recent focus on autologous platelet-rich plasma adjuvant treatment as a promising intervention. Despite the widespread adoption of this surgical technique and the evidence of its non-inferiority compared to other surgical techniques, there has been a lack of consensus in the terminology and methodologies used by various research groups in preparing PCs.

Indeed, the scientific literature contains a plethora of acronyms and definitions for platelet concentrates, leading to a potential challenge in comparing them due to the diverse terminology used. Similarly, during the preparation of platelets, various centrifugation methods are employed, including single or double spins, and different speed ranges. This diversity in preparation methods adds to the complexity and leads to the lack of an established threshold concentration for platelet concentrates efficacy, contributing to further confusion in the field.

To streamline the nomenclature of platelet concentrates, based on this review, we propose employing solely three acronyms: PRP, PRP gel and PRF.

To date, no in vitro studies have been conducted to compare the varying efficacy of these three compounds in tissue healing or to assess the quantity and/or diversity of growth factors they release. Therefore, to date, it remains uncertain whether there is a difference in retinal tissue healing based on the timing of the coagulation cascade activation (pre-contact with the damaged tissue in PRF and PRP gel, or post-contact, PRP).
